# A characterization of *L*_3_(4) by its character degree graph and order

**DOI:** 10.1186/s40064-016-1785-5

**Published:** 2016-03-01

**Authors:** Shitian Liu, Yunxia Xie

**Affiliations:** School of Science, Sichuan University of Science and Engineering, Xueyuan Street, Zigong, 643000 Sichuan People’s Republic of China

**Keywords:** Character degree graph, Projective special linear group, Simple group, Character degree, 20C15, 20C33

## Abstract

Let *G* be a finite group. The character degree graph $\Gamma (G)$ of *G* is the graph whose vertices are the prime divisors of character degrees of *G* and two vertices *p* and *q* are joined by an edge if *pq* divides the character degree of *G*. Let $L_n(q)$ be the projective special linear group of degree *n* over a finite field of order *q*. Khosravi et. al. have shown that the simple groups $L_2(p^2)$, and $L_2(p)$ where $p\in \{7,8,11,13,17,19\}$ are characterizable by the degree graphs and their orders. In this paper, we give a characterization of $L_3(4)$ by using the character degree graph and its order.

## Background

In this paper all groups are finite. Let *G* be a finite group and let Irr(*G*) be the set of all irreducible characters of *G*. Denote by ${\mathrm {cd}}(G)=\{\chi (1):\chi \in {\mathrm {Irr}}(G)\}$ the set of character degrees of *G*.

The graph $\Gamma (G)$ is called *character degree graph* whose vertices are the prime divisors of character degrees of the group *G* and two vertices *p* and *q* are joined by an edge if *pq* divides some character degree of *G* (Manz et al. 1988). Khosravi et al. (2015) proved that the group $L_2(p^2)$, where *p* is a prime, is characterizable by its character degree graph and its order. Khosravi et al. (2014) invested the influence of the character degree graph and order of the simple groups of order less than 6000, on the structure of group. Let $L_n(q)$ be the projective special linear group. By Theorem 3.2(1) of White (2006), we know that $\Gamma (L_3(q))$, where $q>2$ is a power of a prime *p*, is complete if and only if *q* is odd and $q-1=2^i3^j$ for some $i\ge 1,j\ge 0$; also we know that $\Gamma (L_3(4))$ has neither an edge between 2 and 3 nor an edge between 2 and 7.

We know from Khosravi et al. (2014), that the linear groups $L_3(2)\cong L_2(7)$ and $L_3(3)$ are characterized by the character degree graphs and their orders. As a continue of this topics, we will prove the following main theorem.

**Main Theorem** Let $L:=L_3(4)$. If *G* is a finite group such that $\Gamma (G)=\Gamma (L)$ and $|G|=|L|$, then $G\cong L$.

We introduce some notation here. Let $S_n$ and $A_n$ be the symmetric and alternating groups of degree *n*, respectively. Let $L_n(q)$ be the special linear group of degree *n* over finite field of order *q*. If $N\unlhd G$ and $\theta \in {\mathrm {Irr}}(N)$, then the inertia group of $\theta$ in *G* is $I_G(\theta )=\{g\in G\mid \theta ^g=\theta \}$. If *n* is an integer and *r* is a prime divisor of *n*, then we write either $n_r=r^a$ or $r^a\Vert n$ if $r^a\mid n$ but $r^{a+1}\not \mid n$. Let *G* be a group and let *r* be a prime, then denote the set of Sylow *r*-subgroups $G_r$ of *G* by ${\mathrm {Syl}}_r(G)$. If *H* is a characteristic subgroup of *G*, we write $H\,{\mathrm {ch}}\,G$. All other notations are standard (see Conway et al. 1985).

## Some preliminary results

In this section, we give some lemmas to prove the main theorem.

### **Lemma 1**

(Isaacs 1994, Theorem 6.5) *Let*$A\unlhd G$*be abelian. Then*$\chi (1)$*divides* |*G* : *A*| *for all*$\chi \in {\mathrm {Irr}}(G)$.

### **Lemma 2**

(Isaacs 1994, Theorem 6.2, 6.8 and 11.29) *Let*$N\unlhd G$ and let $\chi \in {\mathrm {Irr}}(G)$*. Let*$\theta$*be an irreducible constituent of*$\chi _N$*and suppose that*$\theta _1,\ldots ,\theta _t$*are distinct conjugates of*$\theta$*in G. Then*$\chi _N=e\sum \nolimits _{i=1}^t\theta _i$*, where*$e=[\chi _N,\theta ]$*and*$t=|G:I_G(\theta )|$*. Also*$\theta (1)\mid \chi (1)$*and*$\frac{\chi (1)}{\theta (1)}\mid \frac{|G|}{|N|}$.

### **Lemma 3**

(Xu et al. 2014, Lemma 1) *Let G be a non-soluble group. Then G has a normal series*$1\unlhd H\unlhd K\unlhd G$*, such that K/H is a direct product of isomorphic non-abelian simple groups and*$|G/K|\mid |{\mathrm {Out}}(K/H)|$.

### **Lemma 4**

(Xu et al. 2013, Lemma 2) *Let G be a finite soluble group of order*$p_1^{a_1}p_2^{a_2}\ldots p_n^{a_n}$*, where*$p_1,p_2,\ldots , p_n$*are distinct primes. If*$kp_n+1\not \mid p_i^{a_i}$*for each*$i\le n-1$ and $k>0$*, then the Sylow*$p_n$*-subgroup is normal in G*.

We also need the structure of non-abelian simple group whose largest prime divisor is less than 7.

### **Lemma 5**

(Zavarnitsine 2009) *If S is a finite non-abelian simple group such that*$\pi (S)\subseteq \{2,3,5,7\}$*, then S is isomorphic to one of the following simple groups in Table* 1.

**Table 1:** Finite non-abelian simple groups *S* with $\pi (S)\subseteq \{2,3,5,7\}$

S	Order of *S*	Out(*S*)	S	Order of *S*	Out(*S*)
$A_5$	$2^2\cdot 3\cdot 5$	2	$L_2(49)$	$2^4\cdot 3\cdot 5^2\cdot 7^2$	$2^2$
$L_2(7)$	$2^3\cdot 3\cdot 7$	2	$U_3(5)$	$2^4\cdot 3^2\cdot 5^3\cdot 7$	$S_3$
$A_6$	$2^3\cdot 3^2\cdot 5$	$2^2$	$A_9$	$2^6\cdot 3^4\cdot 5\cdot 7$	2
$L_2(8)$	$2^3\cdot 3^2\cdot 7$	3	$J_2$	$2^7\cdot 3^3\cdot 5^2\cdot 7$	2
$A_7$	$2^3\cdot 3^2\cdot 5\cdot 7$	2	$S_6(2)$	$2^9\cdot 3^4\cdot 5\cdot 7$	1
$U_3(3)$	$2^5\cdot 3^3\cdot 7$	2	$A_{10}$	$2^7\cdot 3^4\cdot 5^2\cdot 7$	2
$A_8$	$2^6\cdot 3^2\cdot 5\cdot 7$	2	$U_4(3)$	$2^7\cdot 3^6\cdot 5\cdot 7$	$D_8$
$L_3(4)$	$2^6\cdot 3^2\cdot 5\cdot 7$	$D_{12}$	$S_4(7)$	$2^8\cdot 3^2\cdot 5^2\cdot 7^4$	2
$U_4(2)$	$2^6\cdot 3^4\cdot 5$	2	$O_{8}^+(2)$	$2^{12}\cdot 3^5\cdot 5^2\cdot 7$	$S_3$

## The proof of Main Theorem

In this section, we give the proof of main theorem.

**The proof of Main Theorem**

### *Proof*

We know from Conway et al. (1985, p. 23), that ${\mathrm {cd}}(L_3(4))=\{1, 20,35,45,63,64\}$. So the graph $\Gamma (G)$ is the graph with vertex set $\{2,3,5,7\}$ and the vertices 5 and 7, and the vertices 2 and 7 have no edge. Therefore there is a character $\chi \in {\mathrm {Irr}}(G)$ with $5\cdot 7\mid \chi (1)$.

It is easy to prove that $O_5(G)=1$ and $O_7(G)=1$. In fact, if $O_7(G)\ne 1$, then $O_7(G)$ is a normal abelian Sylow 7-subgroup of *G* of order 7 by hypotheses. Then by Lemma 1, $\chi (1)\mid |G:O_7(G)|$ for all $\chi (1)\in {\mathrm {cd}}(G)$, a contradiction. Similarly we can prove that $O_5(G)=1$.

Suppose first that *G* is soluble. Let $M\ne 1$ be a minimal normal subgroup of *G*. Then *M* is an elementary abelian *p*-group with *p* = 2 or 3. Note that $|G|_p = p$ for *p* = 5, 7 and in $\Gamma (G)$, there is a character $\chi$ of *G* such that $5\cdot 7$ divides $\chi (1)$. Then by Lemma 1, *p* = 2 or 3. So two cases are considered.

1. Let *M* be a 3-group.
  Since there is a character $\chi$ with $\chi (1)=21$, then $|M|=3$. Let *H* / *M* be a Hall subgroup of *G* / *M* of order $2^6\cdot 5\cdot 7$. Then $|G/M:H/M|=3$. It follows that $(G/M)/(L/M)\hookrightarrow S_3$, where $S_3$ is the symmetric group of degree 3 and $L/M={\mathrm {Core}}_{G/M}(H/M):=\bigcap _{gM\in G/M}(H/M)^{gM}$, the core of *H* / *M* in *G* / *M*. So we have $|L/M|\mid 2^6\cdot 5\cdot 7$ and $Q/M\unlhd L/M$, where $Q/M\in {\mathrm {Syl}}_p(L/M)$ with *p* = 5 or 7. Hence since $L\,{\mathrm {ch}}\,G$, $Q\unlhd G$ and so $|Q|=3\cdot p$. Therefore $O_p(G)\ne 1$ with *p* = 5 or 7, a contradiction.
2. Let *M* be a 2-group.
  If $|M|=2^6$, then by Lemma 1, $\chi (1)\mid |G:M|$, a contradiction. Hence $|M|=2^k$ with $1\le k\le 5$. Let *H* / *M* be a Hall subgroup of order $3^2\cdot 5\cdot 7$. Then $|G/M:H/M|=|G:H|=2^k\le 32$.
  2.1. If $1\le k\le 2$, then $G/H_G\hookrightarrow S_{2^k}$ and so $7\mid |H_G|$. Let $Q/M\in {\mathrm {Syl}}_7(H/M)$. Also $|H_G/M|\mid |H/M|=3^2\cdot 5\cdot 7$. If $|H_G/M|<3^2\cdot 5\cdot 7$, then $Q/M\,{\mathrm {ch}}\,H_G/M\unlhd G/M$ and so $Q\unlhd G$. It follows that $G_7$ is normal in *G*, a contradiction. Hence $|H_G/M|=|H/M|=3^2\cdot 5\cdot 7$. By hypotheses, we can choose a character $\chi \in {\mathrm {Irr}}(G)$ with $\chi (1)=35$. Let $\theta \in {\mathrm {Irr}}(H)$ with $e=[\chi _H,\theta ]\ne 0$. Then $35=et\theta (1)$ with $t=|G:I_G(\theta )|$. Since the numbers *e* and *t* are divisors of $|G:H|=2^{6-k}$, then $e=t=1$ and so $\chi _H=\theta$ by Lemma 2. Since $\theta (1)^2=5\cdot 7\cdot 5\cdot 7<|H|=2^k\cdot 3^2\cdot 5\cdot 7$ and $1\le k\le 2$, then *k* = 2 and $|M|=4$, $M\subseteq H$. Let $\eta \in {\mathrm {Irr}}(M)$ such that $e'=[\theta _M,\eta ]\ne 0$. Therefore $35=e't'$ with $t'=|H:I_H(\eta )|$ Also we know that *M* has 4 linear characters and so $t'\le 4$. So $(e',t')=(35,1)$. It follows that $35^2\le [\theta _M,\theta _M]=e'^2t'\le |H:M|=3^2\cdot 5\cdot 7$, a contradiction.
  2.2. If $3\le k\le 5$, then $M\le H_G\le H$. Therefore $\pi (H_G)=\{2,3\}$, $\{2,5\}$, $\{2,7\}$, $\{2,3,5\}$, $\{2,3,7\}$, $\{2,5,7\}$ or $\{2,3,5,7\}$.
    2.2.1. Let $$\pi (H_G)=\{2,3\}$$.
      Since there is no character $\chi$ with $6\mid \chi (1)$ and *M* is abelian, then $|{\mathrm {cd}}(H_G)|=2$ and $3\mid \chi (1)$ for some character $\chi \in {\mathrm {Irr}}(H_G)$. It folows from Isaacs (1994, Theorem 12.5), *G* either has an abelian normal subgroup of index 3 or 9 or is the product of a 3-group *K* and an abelian. If the former, *k* = 4 (otherwise, the Sylow 3-subgroup is normal in $H_G$) and $|K|=3$. It means $3\cdot 2^2\mid |H/H_G|$ and $2\cdot 3\in {\mathrm {cd}}(G)$, a contradiction. If the latter, then $H_G=(Z_3\rtimes Z_3)\times M$ and so $3\in {\mathrm {cd}}(H_G)$. It follows that there is also an character $\chi$ such that $2\cdot 3\mid \chi (1)$, a contradiction.
    2.2.2. Let $$5\in \pi (H_G)$$.
      Let $Q/M\in {\mathrm {Syl}}_5(H_G/M)$. Then $Q\unlhd H_G\,{\mathrm {ch}}\,G$ and so $G_5\unlhd G$, a contradiction.
    2.2.3. Let $$7\in \pi (H_G)$$.
      Let $Q/M\in {\mathrm {Syl}}_7(H_G/M)$. Then $Q\unlhd H_G\,{\mathrm {ch}}\,G$ and so $G_7\unlhd G$, a contradiction.
    2.2.4. Let $$5,7\in \pi (H_G)$$.
      We can rule out this case as Case 2.2.2 or Case 2.2.3.
    2.2.5. $$H_G$$ is a 2-group.
      Then $M=H_G$. If *G* is an elementary abelian, then by Webb (1983, p. 238), ${\mathrm {Aut}}(G)$ is an extension of an elementary abelian *p*-group of rank $\frac{n^2(n-1)}{2}$ by a subgroup of *GL*(*n*, *p*) (the question of what subgroups of *GL*(*n*, *p*), the general linear group of degree *n* over finite field of order *p*, can arise in this way is still far from a solution). We know that $\frac{G}{C_G(M)}=\frac{N_G(M)}{C_G(M)}\lessapprox {\mathrm {Aut}}(M)$. If *k* = 3, then $5\mid |C_G(M)|$ and the Sylow 5-subgroup $G_5$ of *G* is also a Sylow 5-subgroup of $C_G(M)$. So $G_5\unlhd G$, a contradiction. if *k* = 4 or 5, then $7\mid |C_G(M)|$. Similarly we have $G_7\unlhd G$, a contradiction.

Therefore *G* is insoluble and so by Lemma 3, *G* has a normal series $1\unlhd H\unlhd K\unlhd G$, such that *K*/*H* is a direct product of isomorphic non-abelian simple groups and $|G/K|\mid |{\mathrm {Out}}(K/H)|$.

We will prove that $5,7\in \pi (K/H)$. Assume the contrary, then obviously by Kondrat’ev and Mazurov (2000, Lemma 6(d)) and Liu (2015, Lemma 2.13) apply to almost simple groups $K/H\le G/H \le {\mathrm {Aut}}(K/H)$, where *K*/*H* has a disconnected prime graph. We have that $|{\mathrm {Out}}(K/H)|$ is divisible by neither 5 nor 7. If $5,7\mid |H|$, then there is a Hall $\{5,7\}$-subgroup *L* of *H*, then *L* is cyclic and so *L* is abelian. By Lemma 1, $\chi (1)\mid |G:L|$, a contradiction. If 5 divides the order |*H*| but $7\nmid |H|$, then $G_5$ is cyclic and so get a contradiction by Lemma 1. Similarly, $7\nmid |H|$.

Therefore by Lemma 5 and considering group orders, *K*/*H* is isomorphic to one of the simple groups: $A_7$, $A_8$ or $L_3(4)$.

If $K/H\cong A_7$, then $A_7\le G/H\le {\mathrm {Aut}}(A_7)$. If $G/H\cong A_7$, then there is an edge between the vertices 2 and 3 in $\Gamma (G)$, a contradiction since ${\mathrm {cd}}(A_7)=\{1, 6, 10, 14, 15, 21, 35\}$. Similarly, we can rule out when $G/H\cong S_7$.

If $K/H\cong L_3(4)$, then $L_3(4)\le G/H\le {\mathrm {Aut}}(L_3(4))$. If $G/H\cong L_3(4)$, then $H=1$ and so $G\cong L_3(4)$. For the other cases, we rule out by considering their orders.

If $K/H\cong A_8$, then $A_8\le G/H\le S_8$. If $G/H\cong A_8$, then $H=1$ and so $G\cong A_8$. But $\Gamma (L_3(4))$ has no edge between the vertices 2 and 7, a contradiction. If $G/H\cong S_8$, then order consideration rules out.

So *G* is isomorphic to $L_3(4)$.

This completes the proof. $\square$

### **Corollary**

*Let G be a finite group with*${\mathrm {cd}}(G)={\mathrm {cd}}(L_3(4))$*and*$|G|=|L_3(4)|$*, then G is isomorphic to*$L_3(4)$.

### *Proof*

We know from Conway et al. (1985, p. 23), that ${\mathrm {cd}}(L_3(4))=\{1, 20,35,45,63,64\}$. Since $G_7$ is a Sylow 7-subgroup of *G* with order 7, then $O_7(G)=1$. In fact, if $O_7(G)\ne 1$, then there is a character $\chi$ such that $\chi (1)=70$. So $\chi (1)\mid |G:O_7(G)|$ by Lemma 1. Similarly, $O_5(G)=1$.

Let *G* be a soluble and *M* be a normal minimal subgroup of *G*. Then *M* is an elementary abelian *p*-group. From above arguments, we have *p* = 2, 3. If *p* = 2, then $|M|\ge 2$ and since *M* is abelian, there is no character $\chi$ such that $64\mid \chi (1)$, a contradiction. If *p* = 3, then similarly, there is no character $\chi$ such that $9\mid \chi (1)$, a contradiction.

Therefore *G* is insoluble and so by Lemma 5, *G* has a normal series $1\unlhd H\unlhd K\unlhd G$, such that *K*/*H* is a direct product of isomorphic non-abelian simple groups and $|G/K|\mid |{\mathrm {Out}}(K/H)|$. By Kondrat’ev and Mazurov (2000, Lemma 6(d)) and Liu (2015, Lemma 2.13), $|{\mathrm {Out}}(K/H)|$ is divisible by neither 5 nor 7. Also $5,7\not \mid |H|$ since $O_5(G)=1=O_7(G)$. Hence *K*/*H* is isomorphic to $A_7$, $A_8$ or $L_3(4)$. If $K/H\cong A_7$, then $\Gamma (G)$ is complete, a contradiction since $\Gamma (A_7)$ is complete. If $K/H\cong L_3(4)$, then $G\cong L_3(4)$, the desired result. If $K/H\cong A_8$, then $G\cong A_8$, a contradiction since the vertices 2 and 7 are joined by an edge.

This completes the proof. $\square$

## Conclusion

The projective special linear group $L_3(4)$ can be characterized by the character degree graph and its order. Also we get that $L_3(4)$ is characterized by its order and character degrees.
